# Thyroid and COVID-19: a review on pathophysiological, clinical and organizational aspects

**DOI:** 10.1007/s40618-021-01554-z

**Published:** 2021-03-25

**Authors:** G. Lisco, A. De Tullio, E. Jirillo, V. A. Giagulli, G. De Pergola, E. Guastamacchia, V. Triggiani

**Affiliations:** 1 Interdisciplinary Department of Medicine, Section of Internal Medicine, Geriatrics, Endocrinology and Rare Diseases, School of Medicine, University of Bari “Aldo Moro”, Bari, Apulia Italy; 2 Department of Basic Medical Science, Neuroscience and Sensory Organs, University of Bari Aldo Moro, Bari, Apulia Italy; 3 Department of Biomedical Sciences and Human Oncology, Section of Internal Medicine and Clinical Oncology, University of Bari Aldo Moro, Bari, Apulia Italy

**Keywords:** SARS-CoV-2, COVID-19, Subclinical thyroiditis, Chronic lymphocytic thyroiditis, Hypothyroidism, Hyperthyroidism, Graves’ disease, Thyroid nodules, Pandemic

## Abstract

**Background:**

Thyroid dysfunction has been observed in patients with COVID-19, and endocrinologists are requested to understand this clinical issue. Pandemic-related restrictions and reorganization of healthcare services may affect thyroid disease management.

**Objective and methods:**

To analyze and discuss the relationship between COVID-19 and thyroid diseases from several perspectives. PubMed/MEDLINE, Google Scholar, Scopus, ClinicalTrial.gov were searched for this purpose by using free text words and medical subject headings as follows: “sars cov 2”, “covid 19”, “subacute thyroiditis”, “atypical thyroiditis”, “chronic thyroiditis”, “hashimoto’s thyroiditis”, “graves’ disease”, “thyroid nodule”, “differentiated thyroid cancer”, “medullary thyroid cancer”, “methimazole”, “levothyroxine”, “multikinase inhibitor”, “remdesivir”, “tocilizumab”. Data were collected, analyzed, and discussed to answer the following clinical questions: “What evidence suggests that COVID-19 may induce detrimental consequences on thyroid function?"; "Could previous or concomitant thyroid diseases deteriorate the prognosis of COVID-19 once the infection has occurred?”; “Could medical management of thyroid diseases influence the clinical course of COVID-19?”; “Does medical management of COVID-19 interfere with thyroid function?”; “Are there defined strategies to better manage endocrine diseases despite restrictive measures and in-hospital and ambulatory activities reorganizations?”.

**Results:**

SARS-CoV-2 may induce thyroid dysfunction that is usually reversible, including subclinical and atypical thyroiditis. Patients with baseline thyroid diseases are not at higher risk of contracting or transmitting SARS-CoV-2, and baseline thyroid dysfunction does not foster a worse progression of COVID-19. However, it is unclear whether low levels of free triiodothyronine, observed in seriously ill patients with COVID-19, may worsen the disease's clinical progression and, consequently, if triiodothyronine supplementation could be a tool for reducing this burden. Glucocorticoids and heparin may affect thyroid hormone secretion and measurement, respectively, leading to possible misdiagnosis of thyroid dysfunction in severe cases of COVID-19. High-risk thyroid nodules require a fine-needle aspiration without relevant delay, whereas other non-urgent diagnostic procedures and therapeutic interventions should be postponed.

**Discussion:**

Currently, we know that SARS-CoV-2 could lead to short-term and reversible thyroid dysfunction, but thyroid diseases seem not to affect the progression of COVID-19. Adequate management of patients with thyroid diseases remains essential during the pandemic, but it could be compromised because of healthcare service restrictions. Endocrine care centers should continuously recognize and classify priority cases for in-person visits and therapeutic procedures. Telemedicine may be a useful tool for managing patients not requiring in-person visits.

## Background

Coronavirus disease 2019 (COVID-19) is a highly transmissible infectious disease caused by the Severe Acute Respiratory Syndrome Virus 2 (SARS-CoV-2), a positive-sense, single strand, enveloped RNA virus belonging to the family of beta-coronaviruses [1, 2]. The disease rapidly spread, leading the World Health Organization General-Director Doctor Tedros Adhanom Ghebreyesus to state the ongoing COVID-19 pandemic on March 11, 2020 [3], when 118,000 worldwide confirmed cases were detected in 114 different nations. In most cases, SARS-CoV-2 infection occurs with mild-to-moderate symptoms [4], but a harmful clinical progression has been described in older men and those with underlying comorbidities [5–9]. COVID-19 was found to affect several organs and systems [10], including the endocrine system [11, 12] with possible short and long-term consequences [13]. For instance, the pituitary-thyroid axis should be considered a susceptible target of SARS-CoV-2, and a direct or indirect pituitary injury has been described as a determining factor of possible secondary hypothyroidism (functional or organic) [14]. In line with these suggestions, thyroid dysfunction could be observed during and after a COVID-19 infection, and, therefore, it is expected that some new-onset or recurrent thyroid dysfunctions could be attributable to a recent SARS-CoV-2 infection. Moreover, a pre-existing or new-onset thyroid hormone imbalance, such as the low T3 syndrome, could be associated with the disease severity in COVID-19 [15]. On the other hand, healthcare services have been reorganized, and access to healthcare facilities is restricted to dealing with epidemiological difficulties. As a consequence, thyroid disease management may be potentially affected. Given these assumptions, the authors emphasized some clinical questions related to thyroid diseases/dysfunctions and COVID-19. A point-to-point discussion was carried-out focusing on pre-specified clinical queries as follows: “What evidence suggests that COVID-19 may induce detrimental consequences on thyroid function?”; “Could previous or concomitant thyroid diseases deteriorate the prognosis of COVID-19 once the infection has occurred?”; “Could medical management of thyroid diseases influence the clinical course of COVID-19?”; “Does medical management of COVID-19 interfere with thyroid function?”; “Are there defined strategies to better manage endocrine diseases despite restrictive measures and in-hospital and ambulatory activities reorganizations?”. For this purpose, PubMed/MEDLINE, Google Scholar, Scopus, ClinicalTrial.gov were searched by using free text words and medical subject headings related to “sars cov 2”, “covid 19”, “subacute thyroiditis”, “atypical thyroiditis”, “chronic thyroiditis”, “hashimoto’s thyroiditis”, “graves’ disease”, “thyroid nodule”, “differentiated thyroid cancer”, “medullary thyroid cancer”, “methimazole”, “levothyroxine”, “multikinase inhibitor”, “remdesivir”, “tocilizumab”. Case reports, original articles, randomized controlled trials, reviews, and meta-analysis written in English were analyzed, selected, and discussed.

## Results

### Biochemical and immunological relationship between SARS-CoV-2 and thyroid gland

**Fig. 1:**
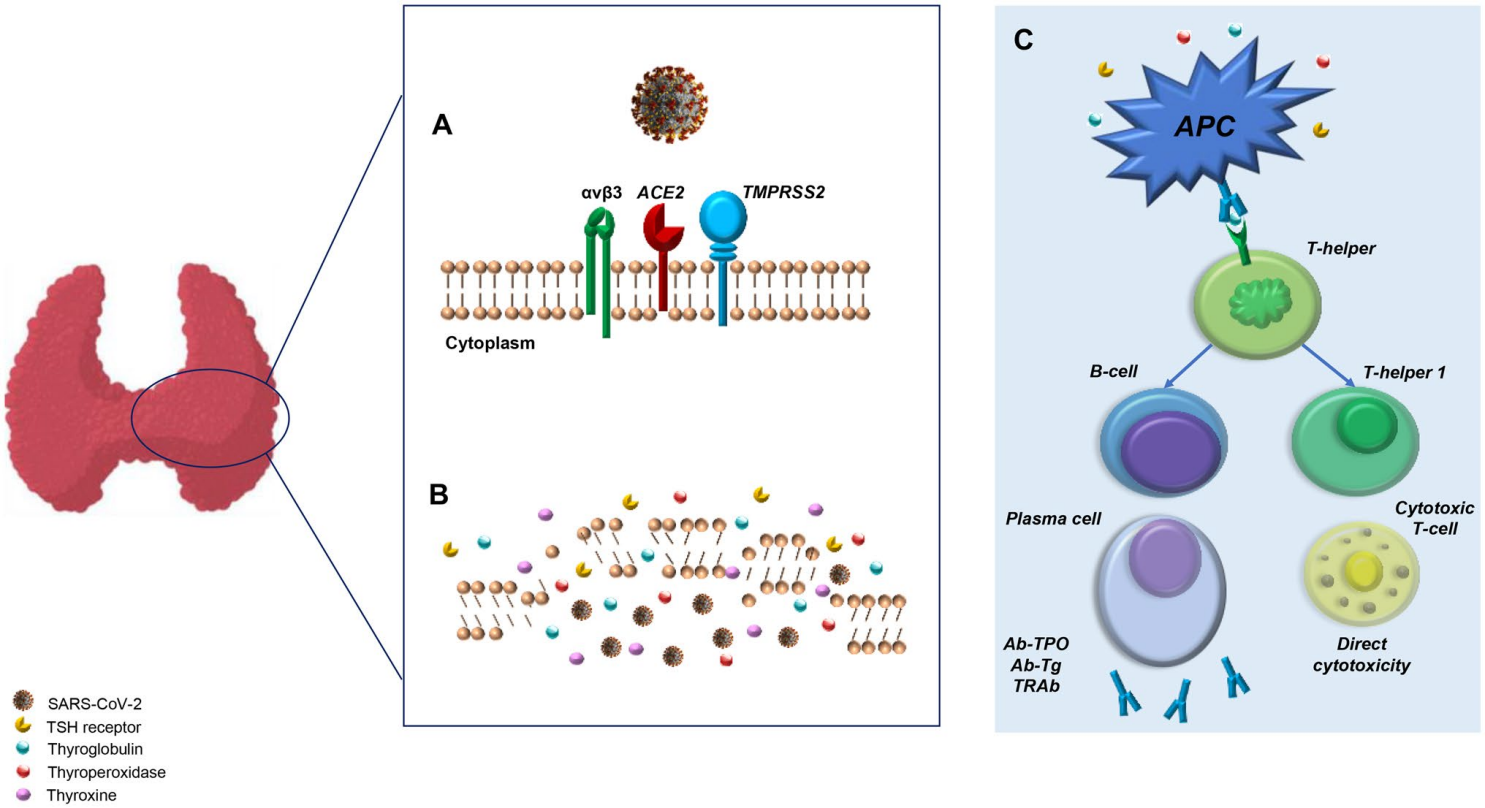
Simplified mechanism of thyroid injury in COVID-19. **a** SARS-CoV-2 internalization into thyrocyte; **b** viral shedding with systemic spread of viral progeny, thyroxine, thyroglobulin, thyroperoxidase, and TSH receptor (acute and subacute thyroiditis); **c** immune-processing of thyroid antigens by antigen-presenting cells (APCs) and consequent activation (in predisposed individuals) of autoreactive lymphocytes (Hashimoto’s thyroiditis, Graves’ disease)

Angiotensin-converting enzyme 2 (ACE2) and transmembrane protease serine 2 (TMPRSS2) are essentially involved in SARS-CoV-2 internalization into host cells playing a relevant role in the pathogenesis of COVID-19 in several species, including humans [16]. These receptors are expressed on a broad range of tissue. The highest levels of ACE2 expression and activity were found at the small intestine, kidney, heart, salivary glands, testicles, and thyroid, whereas lower levels were observed in the brain, skin, pituitary, and skeletal muscles [17, 18]. Thyroid follicular cells express ACE2 as suggested by direct molecular analysis of surgical samples of thyroid tissue [19], leading the gland to be susceptible to SARS-CoV-2 injury once the infection has occurred. In this clinical setting, thyroid damage could result from either a direct or immune-mediated injury [20] (Fig. 1). Integrin αvβ3 is known to recognize and bind Arg-Gly-Asp (RGD) and Lys-Gly-Asp (KGD) motifs localized into the molecular structure of both ACE2 and spike protein of SARS-CoV and SARS-CoV-2, possibly playing a role in antagonizing SARS-CoV-2 binding to ACE2 and consequently reducing SARS-CoV-2 entry into host cells [21]. Since SARS-CoV-2 may alternatively interact with integrin αvβ3, Sigrist et al. hypothesized that this binding might facilitate SARS-CoV-2 internalization into host cells as an alternative pathway respective to ACE2 [22]. According to this point of view, another pathophysiological scenario may have prospected. Indeed, thyroid hormones may bind the integrin receptors on the cell surface, thus, activating signal pathways inside the cell and regulating the transcription of genes involved in anti-apoptotic, angiogenetic properties, and ultimately, supporting cell proliferation [23]. There is evidence that levothyroxine could foster chemo- and radio-resistance and the progression of some thyroid malignancy by activating a second pathway, namely via integrin αvβ3 [24, 25]. Furthermore, levothyroxine may increase the expression of integrin αvβ3 on the cell surface; therefore, T4 may enhance to a more extent SARS-CoV-2 internalization, possibly worsening the prognosis in case of COVID-19 [26]. However, this hypothesis should be confirmed before making conclusions.

Immune response pathways in COVID-19 have recently been reviewed, showing a relevant role of CD4 + and CD8 + T cells by targeting different targets of SARS-CoV-2 to contrast the infection while persisting in the resolution phase of the disease [27]. Additionally, an increase in T-helper (h) 17 and a lower T-regulatory-to-T-h 17 ratio related to high serum levels of interleukin (IL)-6 may contribute to an exaggerated cytokine release, frequently observed in patients with a severe form of the disease [28]. Several cytokines and chemokines, such as IL-1 beta, IL-2, IL-4, IL-6, IL-8, IL-17, IL-22, Tumor Necrosis Factor (TNF) alpha, interferon (IFN) gamma, granulocyte colony-stimulating factor, IFN-gamma induced protein 10 and monocyte chemoattractant protein 1, might additionally play a role in the pathogenesis of COVID-19, especially for the severe form of the disease [29–34]. Given such an immune system imbalance, patients with thyroid autoimmune diseases might undergo a worse clinical course of COVID-19 due to higher baseline levels of serum IL-6 and TNF alpha compared to healthy individuals. On the other hand, SARS-CoV-2 may break immunotolerance in predisposed patients, leading to new onset of immune-mediated thyroiditis, exacerbating a previous thyroid disease, or inducing a recurrence [35–37].

### What evidence suggests that COVID-19 may induce detrimental consequences on thyroid function?

As previously demonstrated for SARS-CoV, also SARS-CoV-2 may lead to thyroid dysfunction [38]. In March 2020, the first case of subacute thyroiditis diagnosed in an 18-year-old woman has been described [39]. The patient was admitted to the hospital with typical signs and symptoms of viral thyroiditis occurring 15 days after a SARS-CoV-2–positive oropharyngeal swab within the context of a mild COVID-19 case [39]. After that, several other cases have been reported worldwide [40–42]. Subacute thyroiditis symptoms occurred 16–36 days after the resolution of COVID-19 [43, 44]. A prompt response to oral prednisone usually occurs, and euthyroidism returns after a few weeks of treatment [44–46].

A cases of Hashimoto’s thyroiditis (HT) with subclinical hypothyroidism in a 45-year-old Chinese man [47] and a myxedema coma in a 69-year-old woman fatality-case have also been described [48]. Thyroid autoimmunity was certainly a pre-existing and undiagnosed condition, but SARS-CoV-2 probably exacerbated the basal condition in these patients. Persistent hypothyroidism, mostly due to HT, has been described in 7% of severe acute respiratory syndrome coronavirus survivors and these data suggest that coronaviruses may have a potential for inducing long-term thyroid dysfunction [49]. In a cohort of 191 mild-to-moderate confirmed COVID-19 cases, thyroid dysfunction has been observed in around 15% of them [50]. Patients with subnormal TSH levels compared to those with normal TSH levels (0.21 mUI/L vs. 1.2 mUI/L) had a more frequent fever (89 vs. 59%, p 0.03) and a lower SARS-Cov-2 cycle threshold value at polymerase chain reaction (20.9 vs. 26.3, p 0.01) suggestive to a higher viral load [50]. In another retrospective study among 50 confirmed COVID-19 patients, more than half of them (56%) had transiently subnormal TSH levels, and those with lower TSH values had poor prognosis [51]. The prevalence of thyroid autoimmunity appeared to be similar or slighter higher [50, 52] than the general population [53, 54]. Considering that autoimmune conditions have been associated to SARS-CoV-2 infection [55], and that viral infection have demonstrated to trigger autoimmune thyroiditis [56], it remains unclear whether thyroid dysfunction occurring in COVID-19 patients could be related to thyroid autoimmunity.

The relationship between severe COVID-19 and the low T3 syndrome is mostly attributable to systemic inflammation [57]. It should be considered that high levels of IL-6 are related to a poorer prognosis of COVID-19 patients, mostly due to a much extent of respiratory involvement [58–60]. IL-6 seems to suppress the production of free levothyroxine (fT4) and free triiodothyronine (fT3) and is implicated in the pathogenesis of the low T3 syndrome [61, 62]. Muller et al. analyzed thyroid dysfunction in critically ill patients in relation to the presence or absence of COVID-19 by comparing hospitalized patients’ data of 2019 to 2020 [52]. After excluding baseline thyroid dysfunction, the results showed lower serum TSH concentration, and higher C-reactive protein levels in COVID-19 (2020) than non-COVID-19 (2019) patients [52]. Moreover, serum fT4 levels were higher, and fT3 were similar in COVID-19 than non-COVID-19 critically ill individuals due to a possible overlapping between a low T3 syndrome and thyroxine toxicosis, attributable to a painless (atypical) subacute thyroiditis [52].

In a retrospective study assessing the prevalence of thyroid dysfunction in confirmed COVID-19 cases, the prevalence of thyrotoxicosis and hypothyroidism was found in 20% and 5%, respectively [63]. Thyrotoxicosis was associated with a more significant hospital stay and a higher in-hospital mortality rate [63]. IL-6 was inversely related to TSH levels, and consequently, thyrotoxicosis resulted in higher concentrations of IL-6 (OR 3.25) [63]. Conversely, fT3 levels appeared to be inversely related to the severity of systemic inflammation [49]. In most cases, the TSH and fT3 levels dropped during the acute phase and remained low during convalescence, but in another longitudinal observational study recruiting 456 patients (334 were admitted to hospital for COVID-19), 86.6% of them were euthyroid, and no cases of overt thyrotoxicosis were diagnosed [64]. TSH and fT4 concentrations at admission were lower than baseline but normalized during the convalescence. A possible drop in TSH levels could be attributable to a blunt pituitary secretion of the thyrotropin due to either a direct and indirect pituitary injury related to SARS-CoV-2 [14, 64]. Altogether these results suggest that thyroid dysfunction may occur, transiently, in COVID-19 patients and could be related to the severity of inflammation. Specific trials are required to clarify the long-term consequences of SARS-CoV-2 infection on thyroid function.

### Could previous or concomitant thyroid diseases deteriorate the prognosis of COVID-19 once the infection has occurred?

A meta-analysis of 8 observational studies found that the frequency of thyroid diseases was higher in COVID-19 patients with poorer prognosis (HR 2.48) [37]; however, some limitations may have affected the result, including study selection methods and a restricted number of cases. The role of hypothyroidism as a possible risk factor for poor prognosis in COVID-19 patients has been investigated more in detail in a retrospective study from New York City [65]. Data were collected from an electronic medical database that included patients with a laboratory-confirmed COVID-19 on a nasopharyngeal swab. Among 3,703 COVID-19 positive patients, 251 (6.8%) had pre-existing hypothyroidism, including those who had received a diagnosis of hypothyroidism and those on levothyroxine treatment. Patients with hypothyroidism compared to those without hypothyroidism were more frequently female (69 vs. 43%, *p* < 0.001), mostly non-Hispanic white (45 vs. 26%, *p* < 0.001), and had more than two underlying comorbidities, such as overweight-obesity syndrome, arterial hypertension, and diabetes mellitus (68 vs. 53%, *p* < 0.001). Despite these differences, a pre-existing hypothyroidism per se was found not to affect the prognosis of COVID-19 [65] even if further investigation needed to evaluate this risk concerning the baseline levels of hypothyroidism control (i.e., TSH and fT4).

A strict relationship between the severity of systemic inflammation, particularly IL-6, and hyperthyroidism was found, and COVID-19 patients with hyperthyroidism and thyrotoxicosis displayed poor prognosis and more extended hospital stay compared to euthyroid patients [63]. IL-6 may induce either the onset or relapse of hyperthyroidism in GD as reported in two case reports [66], and GD relapse cases have been described in convalescent patients, too [67]. Although GD per se does not increase the risk of contracting COVID-19, SARS-CoV-2 infection in GD may disrupt the euthyroidism and make necessary a prompt recognition of hyperthyroidism recurrent/relapse to limit burdens [65]. Uncontrolled hyperthyroidism leads to adverse cardiovascular effects, including cardiac arrhythmias, hemodynamic instability, myocardial ischemia [69], and rises oxidative stress [70], and promotes hypercoagulative-hypofibrinolytic balance [71].

Despite patients with head and neck malignancies had a great chance of poor prognosis in the case of COVID-19, this relationship is still not completely understood for those with thyroid cancer [72]. Surgery and post-surgery phases are known to increase stress, possibly eliciting the pituitary-adrenal axis and catecholamine releases with a consequent and passing suppression of the immune system. Nonetheless, these pathophysiological changes should not be considered risk factors for contracting SARS-CoV-2 or developing a severe clinical progression once infected [72].

### Could medical management of thyroid diseases influence the clinical course of COVID-19?

In seriously ill COVID-19 patients, serum fT3 concentrations were lower than expected and independently predicted all-cause mortality in this cluster of patients [73]. The low T3 syndrome is commonly observed in several acute and chronic clinical conditions such as in sepsis [74], myocardial infarction [75], and chronic heart failure [76, 77], and is the result of an adaptive mechanism whose severity is related to the levels of systemic inflammation [78], serum circulating cortisol, and disturbance of peripheral thyroid hormone transport and metabolism [79]. Normal circulating T3 levels are essential to maintain myocardial tropism [80] and anti-inflammatory effects by reducing peripheral immune cells’ recruitment and attenuating the immune system hyperactivation in response to endotoxemia [81]. T3 also reduces macrophage responsiveness to IL-6, suggesting a potential role of T3 replacement in contrasting an exaggerated systemic inflammation, innate immune response, and ultimately cytokine storm [82]. According to this point of view, the low T3 syndrome per se may affect the prognosis of COVID-19 [83] patients as similarly observed in other clinical scenarios [84–87]. The results of phase II, multicenter, prospective, randomized, double-blind, placebo-controlled trial would clarify the effects of T3 replacement in this setting [88].

Patients with thyroid disorders are equally at risk of contracting the infection as the general population; therefore, a subset of them would be affected by COVID-19 and should be managed according to the disease’s clinical progression. The remaining are likely to be constrained by healthcare services' restrictions for coping with epidemiological conditions. It is necessary to ensure adequate access to cure and satisfactory monitoring methods at a distance. Liothyronine is currently approved to treat hypothyroidism and myxedema coma (intravenously) [89]. Despite a rationale for clinical use in selected cases, either alone or combined with levothyroxine [90], T3 replacement therapy has some drawbacks, including pharmacodynamic and pharmacokinetic characteristics and possible adverse effects, primarily on the cardiovascular system. Oral levothyroxine is a handy and safe medication for replacing hypothyroidism regardless of etiology, and antithyroid medications (ATDs) remain the favored treatment of new-onset hyperthyroidism, especially in GD [91]. A long-term low-dose ATDs therapy is a valuable alternative to radioiodine or thyroidectomy in patients with a persistent or recurrent disease [92], considering pandemic-related limitations [93]. A block and replace regimen could be more appropriate to avoid frequent thyroid function assessments and recurrent in-person visits for therapy adjustments [93]. Agranulocytosis, a rare but serious adverse event, usually occurs within three months after the start of treatment, and the risk is dose-dependent for methimazole (usually > 25–30 mg/day) but not propylthiouracil [94]. In case of signs and symptoms of infection (i.e., respiratory) suggestive for agranulocytosis, a white blood cell count should be promptly obtained, and ATDs should be withdrawn for restoring normal white blood cell count [94]. The management of this complication could be critical because of possible symptoms of misinterpretation (fever, pharyngodynia), and restricted access to healthcare facilities for consultation, diagnosis, and alternative cure.

Target therapies, including multikinase inhibitors and mammalian target of rapamycin inhibitors, have a role in treating advanced thyroid cancers [95–100]. Some kinase inhibitors, including sorafenib, sunitinib, and vandetanib, were used against other viral agents, including coronaviruses, and their efficacy as therapeutic instruments in COVID-19 are currently under investigation [101]. On the other hand, the management of some cancer therapeutics may be a challenge during the pandemic considering possible adverse effects that may foster an undesirable progression of COVID-19 [102]. Reports about the safety and efficacy of multikinase inhibitors for treating advanced thyroid malignancies during the COVID-19 pandemic are currently lacking. Convincing conclusions are difficult to formulate, and starting, continuing, or withdrawing these medications should be carefully personalized possibly after an adequate consultation within a multidisciplinary team for a better assessment of the risk–benefit ratio [68].

### Does medical management of COVID-19 interfere with thyroid function?

The milestones of treatment of COVID-19 remain the targeting of virus and host response by using different strategies, such as antivirals, monoclonal antibodies, anti-inflammatory agents, immune-modulative target therapies [103]. According to the disease's severity [104], different therapeutic approaches are required aiming to reduce the length of transmissibility and the severity of symptoms [105]. As an example, circulatory and ventilatory support and anti-thrombotic therapy could be necessary for seriously ill patients [103]. Bamlanivimab [106] and Casirivimab/Imdevimab [107], neutralizing monoclonal antibodies targeting SARS-CoV-2 spike protein and consequently tackling viral internalization into host’s cells, received the approval for clinical use in patients tested positive within ten days and with a mild or even moderate COVID-19 symptoms, but at higher risk of disease progression due to underlying comorbidities or advanced age. Other clinical trials are currently ongoing in order to investigate the role of several medications for preventing the infection or improving the prognosis of COVID-19 in different clinical scenarios, such as vitamin D (NCT04535791 [108]; NCT04366908 [109]), ascorbic acid (NCT04264533) [110], hydroxychloroquine (NCT04590274) [111]. Some antiviral therapies received approval for emergency use against SARS-CoV-2 or may have a rationale considering their mechanisms of action, including interference with viral internalization (i.e., Umifenovir [112]); RNA-polymerase inhibition (Ribavirin [113], Remdesivir [114]); protease inhibition, including p3-chymotrypsin-like protease (i.e., Favipiravir [115]; Lopinavir/Ritonavir [116]; Sofosbuvir/daclatasvir [117]). These medications have been demonstrated to reduce hospital stay, and accelerate recovery time from disease, especially in moderate cases; however, their efficacy in severe cases could be significantly different according to the results of different trials [118, 119]. Lopinavir and ritonavir may accelerate the metabolism of levothyroxine, diminishing levothyroxine's therapeutic effect [120–122].

Since the critical role of IL-6 in severe pulmonary injury in COVID-19 [123], humanized monoclonal antibodies targeting the IL-6 receptor subunit alpha may have a therapeutic role [124]. For example, tocilizumab reduced mechanical ventilation risk in inpatients with COVID-19, even if the mortality rate remains high in this setting [125]. Baricitinib is an inhibitor of the Janus kinase—signal transducer and activator of transcription proteins or JAK-STAT pathway that mediates cytokines or growth factors receptor signaling [126]. Baricitinib suppresses the production of type 1 IFNs by dendritic cells and IL-6 from B cells [127]. In a double-blind, randomized, placebo-controlled trial, patients on Baricitinib plus RDV recovered faster than those on RDV plus placebo [128]. Baricitinib, administered for atopic dermatitis, did not induce adverse thyroid events [129]; however, it remains unclear whether the possible onset of thyroid autoimmunity would be attributable to an underlying autoimmune condition than the consequence of an adverse event [130].

Dexamethasone at 6 mg/day (for ten consecutive days) either orally or intravenously administered as an add-on to the standard of care versus standard of care alone demonstrated to reduce the 28-day mortality rate by 17%, more evidently in patients requiring invasive mechanical ventilation (− 36%) and in those on supplemental oxygen (− 18%) [131]. Cumulative data suggested that corticosteroids may be beneficial when administered in critically ill patients [132]. Glucocorticoids interfere with thyroid hormone metabolism at several levels, such as by blocking the peripheral conversion of T4 [133], and impair TSH secretion in a dose-dependent manner [134]. In one observation, a single dose of dexamethasone 8 mg administered intravenously reduced the TSH and fT3 levels by 8 to 47 h after the administration [135]. A high-dose dexamethasone regimen (8 mg/day per 3 consecutive days) decreased baseline T3 serum levels without inhibiting T3 response to TSH stimulation [136]. On the other side, the glucocorticoid metabolism may be enhanced in hyperthyroidism and reduced in hypothyroidism, and circulating thyroxine levels may affect cortisol transport and bioavailability being inversely related to cortisol binding protein concentrations [137]. Furthermore, hyperthyroidism fosters albumin synthesis and secretion, and the contrary is observed in hypothyroidism [138]. Considering that albumin regulates dexamethasone distribution and bioavailability, thyroid dysfunction may affect dexamethasone pharmacokinetic influencing this variable [139].

To date, the mechanism of the so-called COVID-19 coagulopathy is well known and represents a combination of localized pulmonary platelet consumption, low-grade intravascular coagulation and thrombotic microangiopathy, endothelial dysfunction, and systemic inflammation [140]. Coagulopathy was found to be the result of both systemic inflammation and SARS-CoV-2 specific-mechanism via ACE2 inhibition, endothelial injury, and dysfunction, induction of autoimmunity [141]. Low-molecular-weight heparin could play a role in thromboprophylaxis, specifically in severe and seriously ill patients, even if further investigations are needed to clarify this topic [142]. Apart from the anticoagulative effects, low-molecular-weight heparin may induce additional effects, such as direct inhibition of viral entry into host cells by interacting with SARS-CoV-2 spike proteins; inhibition of heparanase activity, hence limiting vascular leakage; neutralization of the biological effect of cytokines; interference with leukocyte trafficking [143]. Fractionated and unfractionated heparin displace thyroid hormone from binding proteins, consequently affecting the measurement of both fT4 and fT3 with different assay platforms, such as equilibrium dialysis, ultracentrifugation, and direct immunoassay [144]. Therefore, thyroid hormones should be interpreted cautiously in patients on low molecular weight heparin for avoiding misdiagnosis.

### Are there defined strategies to better manage endocrine diseases despite restrictive measures and in-hospital and ambulatory activities reorganizations?

During the pandemic, several in-hospital and ambulatory activities have been primarily reorganized, restricted, or even suspended to deal with local epidemiological conditions. The number of thyroid fine-needle aspiration procedures was constrained because of healthcare service restrictions [145]. Thus, the incidence of thyroid carcinomas could be underestimated with potentially detrimental consequences, especially for aggressive malignancies [146]. Strategies of possible management of thyroid diseases during the pandemic are necessary to avoid diagnostic and therapeutic delays in severe diseases and postpone safely deferrable non-urgent visits [147]. Fine needle aspiration with cytological assessment should be carried out in patients with high-risk thyroid nodules without relevant delays [145]. Furthermore, patients requiring surgery for thyroid malignancy should undergo an adequate clinical assessment to prioritize interventions that cannot be postponed [148, 149]. Besides clinical aspects, the management of these patients should also be scheduled considering the local transmission rate of COVID-19 and hospital resources [146]. Based upon these assumptions, a proposed endocrine surgery triaging algorithm aligned with the American College of Surgeons has been proposed. All endocrine surgeries could be deferred in case of a rise in transmission rate, except acute airway urgency [150]. French guidelines recommend managing thyroid surgery by scheduling the interventions as follows: urgent; requiring surgery as soon as possible; semi-urgent (surgery should be performed during the acute phase of the pandemic); high-priority elective surgery (surgery soon after the pandemic crisis); distant elective surgery (surgery should be postponed safely) [151]. For example, a thyroid carcinoma without aggressive histology but with overt signs and symptoms of local aggressiveness should be treated as a semi-urgent condition. Conversely, patients with thyroid carcinoma with a tumor size > 2 cm or those with lymph nodes involvement regardless of the primary neoplasm dimension should be scheduled for a high-priority elective surgery. Uncontrolled hyperthyroidism and benign goiter with manifest compressive symptoms should be scheduled for a semi-urgent surgery [151]. On the other side, surgery for low-risk papillary carcinomas may be postponed safely, and a “wait and see” approach should be preferred in this clinical setting.

Follow-up visits in cases of papillary thyroid cancer with an excellent or indeterminate prognosis may be postponed, according to epidemiological complaints, for a post-operative neck ultrasound. Patients could also be managed through telemedicine [72], as a valuable strategy for improving the standard of care in endocrinology during the pandemic [152], also guaranteeing reasonable patient satisfaction despite restrictions [153]. Social media messaging, email service, telephone consultations, and, where available, virtual digital visits could be considered reliable for educational purposes and to ensure a regular follow-up in patients with stable diseases. Laboratory tests may be sent to endocrine care centers periodically by emails or messages, and clinicians verify treatment effectiveness and carry-out minor therapy adjustments. A phone call may also manage minor medical consultations or explanations, while a virtual digital visit may be a useful tool for direct interaction with patients to assess and prioritize cases requiring in-person evaluation according to the medical history.

Nuclear medicine procedures declined remarkably during the lockdown as the consequences of both restrictive measures to contain the spread of the COVID-19 and risk of transmission among healthcare personnel [154]. For patients, radioactive iodine therapy exerts only mild effects on the immune system and should be administered safely in high-risk differentiated thyroid carcinomas.

Graves’ orbitopathy should be managed according to the disease's severity and activity. Selenium supplementation should be considered in mild cases, while intravenous methylprednisolone is recommended in severe, active, and sight-threatening forms of ophthalmopathy [155]. Intravenous methylprednisolone is generally more effective and safer than oral prednisone [156] but should be administered in healthcare facilities after an accurate exclusion of possible contraindications (active tuberculosis, uncontrolled diabetes mellitus, viral hepatitis) [157]. Since the evident superiority of intravenous over oral glucocorticoids in patients with severe ophthalmopathy has been mostly observed in clinical activity score > 5 [158], oral prednisone administered at home may be a good compromise in patients with moderately active orbitopathy. Orbital surgery should be deferred unless an urgent orbital decompression is mandatory [68]. Ocular manifestations of COVID-19 in patients with GD, including conjunctivitis, may lead to possible diagnostic delay of occurring Graves’ orbitopathy manifestations.

## Discussion and conclusions

Thyroid dysfunction should be considered as a possible manifestation of COVID-19. Therefore, thyroid function assessment in patients with COVID-19 may be considered in the diagnostic work-up, particularly in hospitalized patients. In this cluster of patients, the low T3 syndrome prevalence is expected to be high and is related to the severity of COVID-19. However, it is unclear whether T3 administration could improve prognosis in seriously ill patients, as the efficacy/effectiveness and safety of the supplementation are currently under investigation. It should be considered that a concomitant administration of RDV and ATDs in hospitalized patients with hyperthyroidism may increase the risk of acute liver toxicity. Additionally, possible misinterpretation of thyroid dysfunction could result in patients assuming dexamethasone and heparin as these medications could alter thyroid hormone secretion and measurement acutely.

Asymptomatic or mildly affected COVID-19 patients may be managed at home. A thyroid function check should be carried out if required for treatment adjustment or in case of occurring symptoms of thyroid dysfunction.

Non-infected patients with any thyroid diseases should be punctually managed. In this historical moment, telemedicine may be a useful tool for managing patients necessitating frequent therapy adjustments, recurrent medical consultations, or for classifying and prioritizing patients requiring in-person visits unavoidably, such as thyroid aspiration for high-risk thyroid nodules, severe or active Graves’ orbitopathy, management of new-onset or recurrent thyrotoxicosis, ongoing follow-up of progressing thyroid malignancy (Table 1).

**Table 1:** Clinical consequences of SARS-CoV-2 infection and pandemic-related management  challenges based on background thyroid conditions

Background	Clinical consequences	Management challenges and solutions
No thyroid complaints	Subacute thyroiditis (reversible condition after glucocorticoid treatment) [19]	Recurrent thyroid function checking until euthyroidism has been restored Telemedicine service for data collection and medical prescriptions
Thyroid autoimmunity per se	Possible exacerbation or recurrence due to SARS-CoV-2 infection [66, 67]	Timely update of thyroid function in case of symptoms of new-onset or recurrent hyperthyroidism, especially in home self-isolated patients Telemedicine service for data collection and medical prescriptions
Hypothyroidism per se	Nor additive risks of contracting the infection neither possible progression of COVID-19 [65]	Follow-up through telemedicine Levothyroxine can be assumed safely
Uncontrolled hypothyroidism	Higher levels of systemic inflammation and oxidative stress, hypocoagulative imbalance [71, 159]	Timely recognition in both hospitalized and home self-isolated patients Levothyroxine should be started to restore euthyroidism promptly Posology and route of administration (i.e., liquid formulations through a feeding tube; rectal) of levothyroxine according to underlying clinical conditions
Hyperthyroidism per se	Nor additive risks of contracting the infection neither possible progression of COVID-19 [52]	Follow-up through telemedicine Thionamides as the first choice. A "block and replace" strategy may reduce frequent thyroid function monitoring or medical consultations Fever or pharyngodynia may be acute manifestations of both SARS-CoV-2 infection and agranulocytosis in patients on thionamides Concomitant administration of remdesivir and thionamides may increase acute liver toxicity risk
Uncontrolled hyperthyroidism	Higher background inflammation, hypercoagulative imbalance, cardiac arrhythmias, hemodynamic instability [69–71]	Timely recognition in both hospitalized and home self-isolated patients Thionamides as the first choice for promptly restore euthyroidism. Selective beta-blockers should also be prescribed Fever or pharyngodynia may be acute manifestations of both SARS-CoV-2 infection and agranulocytosis in patients on thionamides Concomitant administration of remdesivir and thionamides may increase acute liver toxicity risk
Graves’ orbitopathy	Nor additive risks of contracting the infection neither possible progression of COVID-19	Possible delay in recognizing and diagnosing Graves' orbitopathy in patients with ophthalmic manifestations of COVID-19 Prioritize severe ophthalmopathy for intravenous methylprednisolone
Thyroid nodules	Nor additive risks of contracting the infection neither possible progression of COVID-19	Possible delay of the cytological definition of high-risk nodules Neck ultrasound could be required to assess/re-assess nodule characteristics Selection of high-risk nodules for undeferrable fine-needle aspiration and cytology
Thyroid malignancies	Thyroid cancer malignancy is not a risk factor for a poorer prognosis of COVID-19	A careful and structured clinical triage is needed for adequately scheduling the management of patients who require interventions with priority Telemedicine as a tool for TSH and thyroglobulin monitoring in patients with an excellent prognosis

## Abbreviations

COVID-19: Coronavirus disease 2019
SARS-CoV-2: Severe Acute Respiratory Syndrome Coronavirus 2
ACE2: Angiotensin-converting enzyme 2
TMPRSS2: Transmembrane protease serine 2
T-h: Lymphocyte T helper
IL-6: Interleukin-6
TNF: Tumor necrosis factor
IFN: Interferon
fT4: Free levothyroxine
fT3: Free triiodothyronine
TSH: Thyroid-Stimulating Hormone
HT: Hashimoto’s thyroiditis
GD: Graves’ disease
ATDs: Antithyroid drugs
VD: Vitamin D
RDV: Remdesivir

## Declarations

### Human and animals participants statements

This article does not contain any studies with human participants or animals performed by any of the authors.
